# Increased fatty acid synthase expression in prostate biopsy cores predicts higher Gleason score in radical prostatectomy specimen

**DOI:** 10.1186/1472-6890-14-3

**Published:** 2014-01-14

**Authors:** Shinsuke Hamada, Akio Horiguchi, Kenji Kuroda, Keiichi Ito, Tomohiko Asano, Kosuke Miyai, Keiichi Iwaya

**Affiliations:** 1 Department of Urology, National Defense Medical College, 3-2 Namiki, Tokorozawa-City, Saitama 359-8513, Japan; 2 Department of Basic Pathology, National Defense Medical College, Tokorozawa-City, Saitama, Japan

**Keywords:** Fatty acid synthase, Prostate cancer, Biopsy core, Gleason score

## Abstract

**Background:**

Fatty acid synthase (FAS) is highly expressed in various types of cancer, and elevated expression of FAS has been suggested to be a predictor of tumor aggressiveness and poor prognosis. We examined whether FAS expression in prostate biopsy cores could predict the pathological characteristics of radical prostatectomy (RP) specimens.

**Methods:**

Paraffin-embedded prostate biopsy cores, obtained from 102 patients who subsequently underwent RP, were immunostained with polyclonal anti-FAS antibody. The staining intensity was categorized into non-staining, weak, moderate, and strong. Tumors with moderate or strong immunostaining were considered to show high FAS expression, and other tumors were considered to show low FAS expression. The relation between the FAS expression status in biopsy cores and pathological parameters in RP specimens was analyzed.

**Results:**

The FAS expression in the biopsy cores of 64 of the 102 tumors (63%) was high, whereas it was low in the biopsy cores of the other 38 tumors (37%). High FAS expression was significantly associated with Gleason Score (GS) ≥ 7 in RP specimens (p< 0.0001). In multivariable logistic regression analyses, GS ≥7 in biopsy cores (p <0.0001), higher preoperative PSA (p = 0.0194), and high FAS expression (p = 0.0004) were independent predictors of GS ≥ 7 in the RP specimen.

**Conclusions:**

Increased FAS expression in prostate biopsy cores could be a novel parameter for predicting higher GS in RP specimens. The treatment strategy for patients with high FAS expression in prostate biopsy cores should be carefully determined.

## Background

Fatty acids are essential constitutions of all biological membrane lipids, and are very important substrates for energy metabolism [1]. The biosynthesis of endogenously synthesized fatty acids is catalyzed by multifunctional homodimeric fatty acid synthase (FAS) [1,2]. FAS is a bio-synthetic enzyme in lipogenesis and the production of long-chain fatty acids from acetyl-coenzyme A (Co A), malonyl-CoA, and nicotinamide adenine dinucleotide phosphate (NADPH) [1-3]. Under normal conditions, FAS converts excess carbohydrates into fatty acids that are then esterified to storage triacylglycerols and provide energy through β-oxidation [1,2]. Except for liver and adipose cells, normal cells in humans have low or undetectable levels of expression and activity of FAS, which is closely regulated by diet, hormones, and growth factors [1,2].

On the other hand, FAS is highly expressed in various types of cancer, and elevated expression of FAS has been linked to tumor aggressiveness and poor prognosis and reduced disease-free survival [4-6]. FAS was first identified as an oncogenic antigen 519 (OA 519) in patients with breast cancer and a markedly poorer prognosis [7]. FAS expression is an indicator of poor prognosis in breast and prostate cancers and is elevated in the blood of cancer patients [3].

However, to the best of our knowledge, the predictive value of FAS expressed in prostate biopsy cores has yet to be clarified as to the clinicopathological parameters of prostate cancer. On the basis of the studies mentioned above, we hypothesized that the expression status of FAS in prostate biopsies might provide valuable information for clinicians. In this study, we performed an immunohistochemical analysis of FAS expression in prostate biopsy cores and a statistical analysis on the relationship between the expression status of FAS prostate biopsy cores and clinicopathological parameters.

## Methods

### Patients

This study included 102 patients (median age 67, range 51–75) who had underwent ultrasonically guided transrectal and/or transperinieal prostate biopsies and radical prostatectomies (RP) for clinically localized prostate cancers at the National Defense Medical College from May 1998 to April 2007. Patient characteristics are listed on Table 1. Clinical T stage was assessed by digital rectal examination and/or transrectal ultrasound in accordance with the 2009 TNM classification. The Gleason grading system for biopsies and surgical specimens was in accordance with the 2005 International Society of Urological Pathology (ISUP 2005) [8]. The positive core rate was defined as the number of positive biopsy cores divided by total number of biopsy cores. The levels of serum PSA were measured on the day before the operation. Patients who received hormonal therapy or radiation therapy prior to RP were excluded from the analysis. The follow-up period calculated from the date of the operation to the date of the last recorded follow-up ranged 1.0 to 149.5 months (median: 73 months). PSA failure was considered to have occurred if PSA value was over 0.2 ng/ml after RP. We obtained informed consent for participation in the study from participants. This study was approved by the National Defense Medical College ethical committee.

**Table 1:** Patient characteristics

**Parameters**	**No. (%)**
Over all	102
Median age (range)	67 (51-75)
Clinical T stage	
cT1c	71 (69.6)
cT2a	17 (16.7)
cT2b	3 (2.9)
cT2c	0 (0)
cT3	11 (10.8)
PSA (ng/ml)	
0-4	2 (2.0)
4-10	58 (56.9)
10-20	25 (24.5)
20-	17 (16.6)
median (range)	8.172 (3.3-61.9)
Total biopsy cores (median)	6 -24 (8)
Positive core rate (%) (median)	5.56 -100 (25)

### Immunohistochemical analyses of FAS expression in prostate biopsy cores

Formalin-fixed, paraffin-embedded biopsy cores, 4 μm thick, were deparaffinized in xylene and rehydrated through graded ethanols. Endogenous peroxidase activity was quenched with DAKO Peroxidase Blocking Reagent (Dako Corporation) for 10 minutes. The slides were incubated in 10% normal goat serum in PBS for 60 minutes at room temperature. After the slides were incubated 60 minutes in a 1:50 dilution of rabbit polyclonal anti-FAS antibody (Immuno-Biological Laboratories Co., Fujioka, Japan) in PBS, the slides were stained by using a Simple Stain Max PO (Nichirei Corporation, Tokyo, Japan) according to the manufacturer’s instructions. Reaction products were visualized by immersing the slides in DAB for 2 minutes. The immunohistochemistry was evaluated independently by 2 observers (K.M. and K.I.) blinded from any clinical data. Whole biopsy slides were evaluated in all cases examined. While comparing with the slides of hematoxylin-eosin stain, only prostatic cancer cells were taken into account for the evaluation of FAS protein expression. Cytoplasmic intensities of FAS immunoreactivity were classified semiquantitatively into the four categories: non-staining (negative), weak (score 1+), moderate (score 2+), and strong (score 3+) [9,10]. When there were heterogeneous intensities of FAS immunoreactivity within examined cases, strongest intensity was adapted as the score of FAS expression in each case. The staining intensity in normal prostate gland was used as an internal control. Any discrepancies between the assessments of the 2 observers were resolved by conferring over a multiviewer microscope. Tumors with 3+ or 2+ immunostaining were considered high, and other tumors were considered low. The Gleason score (GS) in the biopsy core was determined on the same sections.

### Statistical analysis

All statistical analyses were performed by using the JMP 9.0 soft-ware system for Windows (SAS Institute Inc. Cary, NC, USA). A chi-square test was used to assess the association between preoperative and pathological specimen parameters relative to the level of FAS expression. Logistic regression analyses were used for multivariate analyses to predict RP specimen GS ≥7. Receiver operating characteristic (ROC) curves were plotted with the sensitivity (true-positive fraction) on the y-axis versus 1 minus the specificity (false-positive fraction) on the x-axis. The PSA failure rate was calculated and compared by log-rank testing. P values less than 0.05 were considered to indicate statistical significance.

## Results

### Relations between FAS expression in biopsy cores and preoperative parameters

**Figure 1:**
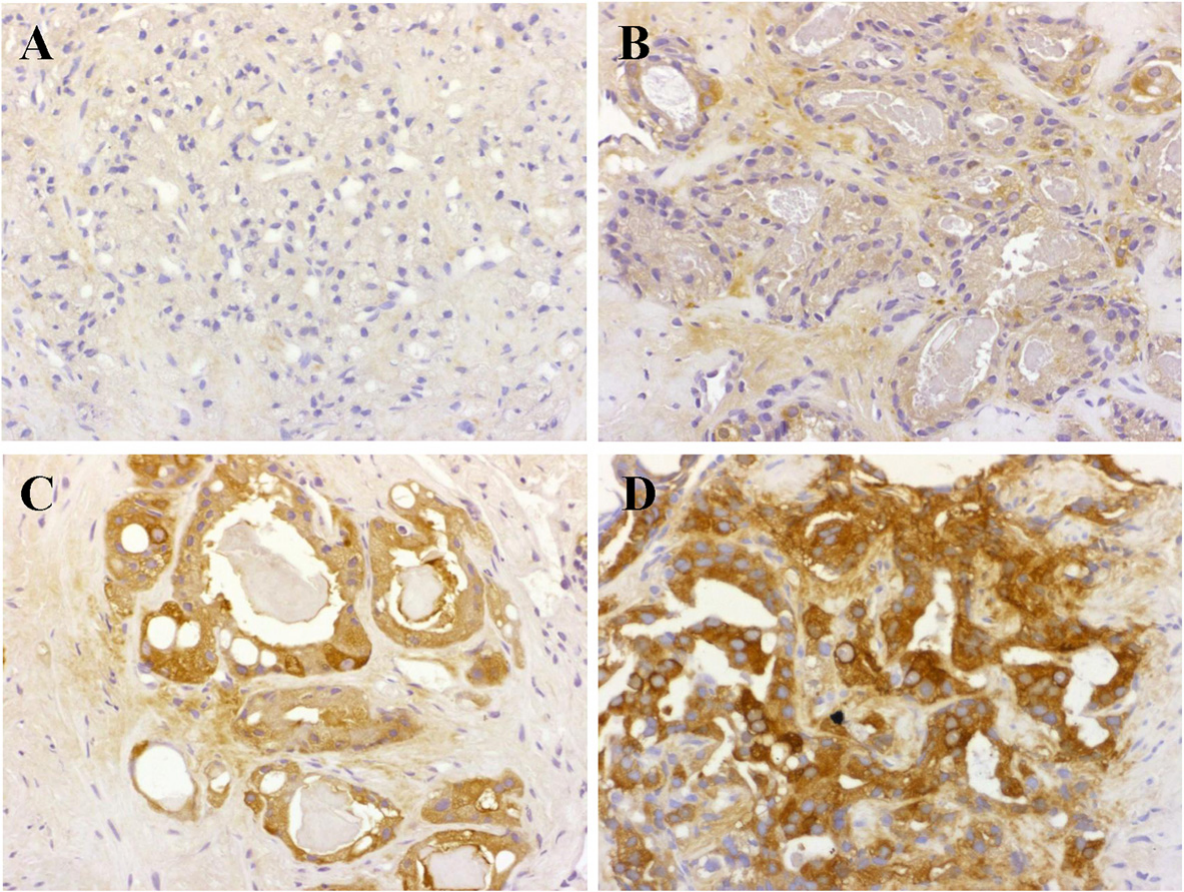
**Representative immunohistochemistry for fatty acid synthase (FAS) in prostatic adenocarcinoma. A**, Case of prostatic adenocarcinoma showing no immunoreactivity for FAS (score 0). **B-D***,* Cases of prostatic adenocarcinoma showing weak **(B)**, moderate **(C)**, and strong **(D)** cytoplasmic immunoreactivity for FAS, and scored as 1+, 2+, and 3+, respectively. Immunoperoxidase stain, original magnification x200

We first evaluated the expression level of FAS in prostate biopsy cores. FAS expression was high in the biopsy cores of 64 of the 102 tumors (63%) and low in the other 38 tumors (37%): 4 (4%) showing negative immunostaining, 34 (33%) showing 1+ immunostaining (weak staining), 44 (43%) showing 2+ immunostaining (moderate staining), 20 (20%) showing 3+ immunostaining (strong staining) (Figure 1). Table 2 lists the p values for the associations between FAS status and preoperative parameters. High FAS expression was significantly associated with clinical T stage (cT2-cT3) (p =0.0381), GS ≥ 7 in the biopsy core (p = 0.0044) and positive core rate > 40% (p = 0.0038). Preoperative parameters including PSA and D’Amico risk classification [11] were not associated with FAS expression status (Table 2).

**Table 2:** FAS expression and preoperative parameters

		**FAS expression**		
**Parameters**	**Patients**	**Low**	**High**	**P value**
Clinical T stage				
cT1c	71	31	40	0.0381
cT2-cT3	31	7	24	
PSA (ng/ml)				
≤ 10	60	24	36	0.4919
10 <	42	14	28	
Needle biopsy Gleason score				
≤ 6	31	18	13	0.0044
7 ≤	71	20	51	
Positive core rate (%)				
≤ 40	72	33	39	0.0038
40 <	30	5	25	
D’Amico risk classification				
Low and Intermediate risk	67	28	39	0.1853
High risk	35	10	25	

### Relations between FAS expression and pathological parameters of RP specimens

Next, we analyzed the association between FAS expression in the biopsy cores and pathological features of RP specimens. High FAS expression was significantly associated with GS ≥7 in the RP specimens (p< 0.0001, Table 3). However, high FAS expression was not significantly associated with any other pathological parameters, including status of extraprostatic extention, lymphatic invasion, microvessel invasion, or perineural invasion. Although high FAS expression in biopsy cores was significantly associated with positive core rate > 40%, it was not associated with maximum tumor diameter in RP specimens.

**Table 3:** FAS expression and pathological parameters

		**FAS expression**		
**Parameters**	**Patients**	**Low**	**High**	**P value**
Gleason score				
≤ 6	23	18	5	< 0.0001
7 ≤	79	20	59	
Extra-prostatic extension				
Positive	45	14	31	0.3002
Negative	56	23	33	
Lymphatic invasion				
Positive	51	18	33	0.6821
Negative	51	20	31	
Microvascular invasion				
Positive	40	13	27	0.4232
Negative	62	25	37	
Perineural invasion				
Positive	71	23	48	0.0978
Negative	30	15	15	
Maximum tumor size				
≤ 10 mm	13	6	7	0.4823
10 mm <	89	32	57	

### High FAS expression in biopsy cores is an independent predictor of GS ≥ 7 in RP specimens

The ability to predict GS in RP specimens using preoperative parameters is quite important for the selection of therapies for localized prostate carcinoma [12,13]. On the basis of our results, we examined whether the FAS expression in biopsy cores could be a novel preoperative predictor of higher GS in the RP specimens. In the univariate analysis, preoperative parameters including clinical T stage, GS ≥7 in biopsy cores, PSA, positive core rate, and high FAS expression in biopsy cores were significantly associated with GS ≥7 in RP specimens. In the multivariable logistic regression analyses, biopsy GS ≥ 7 (p < 0.0001), increasing preoperative serum PSA level (p = 0.0202), and high FAS expression (p = 0.0012) were independent predictors of GS ≥ 7 in RP specimens (Table 4).

**Table 4:** Univariate and multivariate analyses to predict specimen Gleason score ≥ 7

	**Univariate**			**Multivariate**		
	**P value**	**Odds ratio**	**95% CI**	**P value**	**Odds ratio**	**95% CI**
FAS expression in biopsy cores (Low vs. High)	< 0.0001	10.62	3.71-35.65	0.0012	34.15	3.42 - 1145.4
Clinical T stage (cT1 vs. cT2 ≤)	0.0293	3.66	1.13-16.49	0.4890	2.29	0.23 - 32.36
Needle biopsy Gleason score ( ≤ 6 vs. 7≤)	< 0.0001	72.45	17.84-499.7	< 0.0001	228.17	25.37 - 8358.31
PSA (ng/ml)	0.0174	1.09	1.01-1.19	0.0202	1.22	1.03 -1.57
Positive core rate (%) (≤ 40% vs. 40% <)	0.007	5.76	1.53-37.7	0.9106	1.14	0.10 - 10.95
Total biopsy cores (≤ 8 vs. 8 <)	0.4131	1.48	0.58-3.84	0.9423	1.08	0.14 - 8.42

### ROC analyses

**Figure 2:**
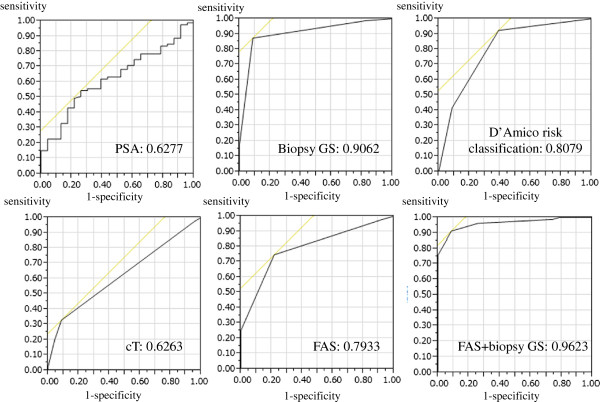
Receiver operating characteristic (ROC) curves and areas under the ROC curves (AUC) of PSA, biopsy Gleason score, D’Amico risk classification, clinical T stage, FAS expression, and needle biopsy Gleason score added to FAS expression for predicting RP specimen Gleason score ≥7.

The area under the ROC curve (AUC) was calculated (Figure 2) as a way to estimate the predictive value of the preoperative parameters for RP specimens with GS ≥7. AUC was 0.6277 for PSA, 0.9062 for needle biopsy GS, 0.6263 for clinical T stage, 0.8079 for D’Amico risk classification, and 0.7933 for FAS expression. In particular, the AUC of the combination of FAS and GS ≥7 in the biopsy cores reached 0.9623.

### FAS expression and the period to PSA failure

**Figure 3:**
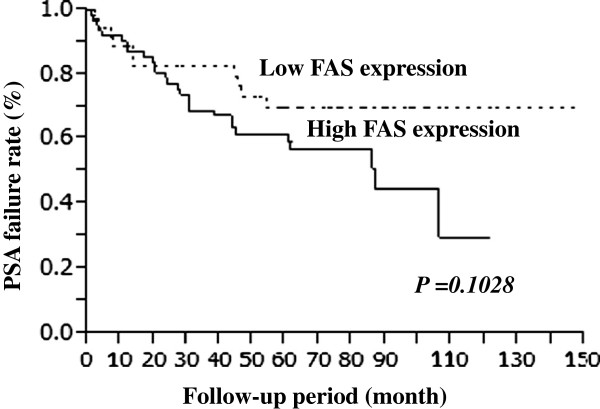
Kaplan-Meier curves for PSA failure rates after RP in patients with high and low FAS expression.

The Kaplan-Meier survival curves for PSA fairure rate is shown in Figure 3, where they are stratified by FAS expression. Although patients with high FAS expression tended to have high PSA failure rates compared with those with low FAS expression, it did not reach statistically significant (p = 0.1028).

## Discussion

Fatty acids are synthesized to provide lipids for energy production via β-oxidation and lipid modification of proteins and membrane formation in cancer cells [1-3]. FAS is highly expressed in many cancers, including thyroid, oral, tongue, esophageal, lung, breast, endometrial, ovarian, colorectal, bladder, hepatocellular, pancreatic and gastric carcinomas, as well as sarcoma, Paget’s disease of the vulva, gastrointestinal stromal tumor, and multiple myeloma [1-3,14-16]. Regarding urological malignancies, increased expression and its association with tumor aggressiveness and poor cancer-specific survival are evident in patients with prostate and kidney cancer [9,17-19].

The relationship between FAS and prostate cancer has been widely investigated. FAS was over-expressed in high grade prostatic epithelial neoplasia (PIN), and FAS expression was found to be possibly one of the earliest events in the development of prostate cancer [9]. FAS expression was also a strong independent predictor of pathologic stage [17]. While FAS expression in benign tissue was reduced after androgen ablation, it persisted in human prostate carcinoma after androgen ablation and was high in 82% of lethal tumors examined by autopsy [18]. Increased FAS expression appeared to increase the risk of death from prostate cancer 4.4-fold [6]. FAS may be used as a prostate cancer marker, and anti-neoplastic therapy based on FAS inhibitions may be an option for chemo prevention or curative treatment of nearly all prostate cancers [9]. These observations suggest that FAS expression status could be an important parameter of tumor aggressiveness and in prostate cancer. Although the mechanism of cell malignant transformation or cell proliferation induced by FAS over expression in malignant tumor is not fully understood, several mechanisms including increasing expression of epidermal growth factor receptor and activation of PI3K/AKT and ERK1/2 signaling pathways, and the modulation and nuclear maturation of the transcription factor sterol regulatory element-binding protein 1c have been reported [1-3].

Most of the studies on FAS expression in prostate cancer examined RP specimens, and a few studies focused on FAS expression in biopsy cores [4,6,9,17,18]. Bandyopadhyay *et al*. and Swinnen *et al*. reported that FAS expression levels in prostate cancer specimens including needle biopsies were correlated with the degree of Gleason grade [6,9]. In our study’s results, high FAS expression in biopsy cores was significantly associated with biopsy cores GS ≥7, which is consistent with previous observations [6,9]. We therefore speculated that the expression status of FAS in the biopsy core could be a novel diagnostic tool for evaluating tumor aggressiveness in prostate cancer. The decision of which treatment to pursue in clinically localized prostate cancer depends on the aggressiveness of the tumor. GS in biopsy cores is one of the most valuable parameters of tumor aggressiveness. The ability to assign a precise GS in the prostate is quite important for selecting appropriate treatments for prostate cancer. However, the GS values in about 40–50% of RP specimens do not correspond with those calculated from needle biopsies [12,13,20]. Various parameters including patient age, PSA, maximum percentage cancer/biopsy cores, the number of biopsy cores and surgical specimen weight have been shown to be useful for precisely predicting GS [12,20] in RP specimens, but none has proven to be definitive. In our study, high FAS expression of biopsy cores was an independent predictor of RP specimens GS≥7 in addition to GS of biopsy cores. In particular, the AUC of the combination of FAS and GS ≥7 in biopsy cores reached 0.9623.

Our studies have some limitations. Although we analyzed the relationship between FAS expression and the period to PSA failure, there was no significant association between the parameters. We consider that the underlying reasons include insufficient numbers of patients and RPs performed by variety of surgeons with different skills. We could not analyze the relationship between nutritional status and FAS expression levels. In recent study, it has been reported that overweight prostate cancer patients tend to have germline single nucleotide polymorphisms (SNPs) in the *FAS* gene and FAS tumor expression, suggesting FAS might play the key role in poor outcome in obese prostate cancer patients [21]. Despite such limitations, our results provide novel information about biopsy cores for precisely predicting GS.

## Conclusions

Increased FAS expression in prostate biopsy cores could be a novel parameter for predicting higher GS in RP specimens. The treatment strategy for patients with high FAS expression in prostate biopsy cores should be carefully determined.

## Abbreviations

FAS: Fatty acid synthase; RP: Radical prostatectomy; GS: Gleason score; ROC: Receiver operating characteristic; AUC: The area under the ROC curve.

## Competing interests

The authors declare that they have no competing interests.

## Authors’ contributions

SH and AH were responsible for drafting the manuscript. KM and KI contributed to immunohistochemistry. KK, KI and TA contributed to analysis and interpretation of data. All authors read and approved the final manuscript.

## Pre-publication history

The pre-publication history for this paper can be accessed here:

http://www.biomedcentral.com/1472-6890/14/3/prepub
