# Erratum: Increased fatty acid synthase expression in prostate biopsy cores predicts higher Gleason score in radical prostatectomy specimen

**DOI:** 10.1186/s12907-015-0007-3

**Published:** 2015-05-13

**Authors:** Shinsuke Hamada, Akio Horiguchi, Kenji Kuroda, Keiichi Ito, Tomohiko Asano, Kosuke Miyai, Keiichi Iwaya

**Affiliations:** Department of Urology, National Defense Medical College, Tokorozawa-City, Saitama Japan; Department of Basic Pathology, National Defense Medical College, Tokorozawa-City, Saitama Japan

## Erratum

We acknowledge that in the published work [1], text in the Methods and Conclusions sections was duplicated from our previous publication, Hamada et al., 2014, Prostate [2] by mistake and we inadvertently failed to include this paper in the reference list. We would like to further clarify the relationship between these manuscripts. In 2012, we had first started to collect and analyze the data from patients with any biopsy Gleason score who underwent radical prostatectomy from 1998 to 2007 for prostate cancer. These data were included in the manuscript published in BMC Clinical Pathology [1]. We then generated another dataset of only patients whose biopsy Gleason score was less than 6 and who underwent radical prostatectomy from 2007 to 2012 (of which 31 were overlapping with dataset from 1999 - 2007), reported in the Prostate paper [2]. At the time of submission, we did not consider that the contents of these papers were similar, and we did not inform the publisher about the other manuscript. We would like to clarify that we consider that the aims and highlights of the two papers are quite different and both results are very important for clinicians. We apologize for any inconvenience caused.
